# Attitudes toward the adoption of eHealth amongst healthcare professionals in trauma surgery – the new digital normal?

**DOI:** 10.1186/s12913-024-11259-7

**Published:** 2024-12-18

**Authors:** Jelle Friso Spierings, Gijs Johan Antoon Willinge, Bas Anne Twigt, Sjoerd Repping, Marike Cornelia Kokke, Ruben van Veen, Detlef van der Velde

**Affiliations:** 1 Department of Trauma surgery, St. Antonius Ziekenhuis Utrecht, Soestwetering 1, Utrecht, 3543 AZ The Netherlands; 2 Department of Trauma Surgery, OLVG, Jan Tooropstraat 164, Amsterdam, 1061 AE The Netherlands; 3 Amsterdam University Medical Centers, Meibergdreef 9, Amsterdam, 1105 AZ the Netherlands

**Keywords:** Adoption, eHealth, COVID-19, Trauma surgery, Orthopaedic surgery

## Abstract

**Background:**

As in many other countries, the Dutch emergency healthcare system is under pressure due to increasing numbers of patients, limited budgets, and constrained (human) resources (TraumaNet AMC 19 May, 2016; Int J Emerg Med 6:41, 2013). eHealth, enlarged by the COVID-19 pandemic, has been advocated to substitute face-to-face care to alleviate the pressure of the burden of care (Ministry of Health Welfare and Sport, 2022; Dutch Society of Hospitals, 2022). In order for eHealth solutions to be adopted in daily practice, is it essential to assess healthcare professionals’ attitudes toward its usefulness. As this is currently lacking, this study explores the use of eHealth in daily practice, opportunities of eHealth, implementation barriers, and desired functions and features amongst healthcare professionals working in Dutch orthopedic surgery and traumatology.

**Methods:**

A cross-sectional, web-based survey among the 605 members of the Dutch Society of Trauma Surgery and related healthcare professionals on the attitudes towards eHealth in daily practice was performed between November 4, 2021, and March 31, 2022. The survey consisted of five sections with 42 questions, including close-ended questions, multiple-choice questions, 5-point Likert Scales, Visual Analogue Scales, and free-text questions.

**Results:**

Of the 111 responding healthcare professionals, 59/111 (53%) were male, and the median age was 40 years (IQR 26 to 67). Almost all participants owned smartphones (109/111, 98.2%). Most participants reported that the COVID-19 pandemic had influenced their attitude towards the usefulness of eHealth positively (80/111, 72%). Most participants (59%) would use a digital alternative instead of face-to-face follow-up if proven a safe technology, and expect that 64% of all patients would prefer a digital option. Most healthcare professionals stated that eHealth could reduce healthcare costs (94/111, 85%) and improve patient satisfaction (81/111, 73%) but is hindered most by a lack of financial support during implementation (57/111, 51%), followed by complex laws and regulations (54/111, 49%).

**Discussion:**

Results of this cross-sectional survey show that attitudes of orthopedic surgery or traumatology-related healthcare professionals toward the usefulness of eHealth are positive and may have increased during the COVID-19 pandemic. Even though healthcare professionals believe eHealth could reduce costs and improve patient satisfaction, daily clinical use remains low possibly due to a lack of long-term and short-term financial support and complex laws and regulations.

**Supplementary Information:**

The online version contains supplementary material available at 10.1186/s12913-024-11259-7.

## Introduction

The Dutch emergency healthcare system is under pressure due to an increasing number of patients requiring in-hospital care, limited budgets, and constrained (human) resources [1, 2]. Digital care alternatives, also known as eHealth, have been advocated to substitute face-to-face care, to alleviate the pressure of the increasing burden of care [3, 4]. Even though the term eHealth was already introduced in 1999, the digital transformation of healthcare remains notoriously slow compared to other industries (e.g., the smartphone industry) [5].

eHealth is defined as an emerging field in the intersection of medical informatics, public health and business, referring to health services and information delivered or enhanced through the Internet and related technologies. In a broader sense, the term characterizes not only a technical development, but also a state-of-mind, a way of thinking, an attitude, and a commitment for networked, global thinking, to improve health care locally, regionally, and worldwide by using information and communication technology [5]. eHealth is considered a part of digital health, which has been defined as the use of information and communication technologies in medicine and other health professions to manage illnesses and health risks and to promote wellness [6].

The slow adoption of eHealth particularly applies to surgical departments, where patients with acute injuries or diseases and relatively short follow-up periods (e.g., fractures or appendicitis) are treated [7, 8]. This study focuses on the use of eHealth in Dutch orthopedic trauma surgery after the start of the COVID-19 pandemic. Since the COVID-19 pandemic, there is an increase of publications regarding self-care applications, timely information, and telemonitoring in (Dutch) orthopedic trauma surgery [9–12]. This increase correlates with the international literature, describing a similar increase use of eHealth in orthopedic surgery, particularly in postoperative and perioperative care for major surgery to cope with shorter perioperative admissions and reduced outpatient care. An increase from 62 articles in 2018 to 273 articles in 2021 has been reported [13]. However, actual use, required functions, and motives for non-use remain underreported for the Dutch population and internationally [14].

The limited implementation of eHealth in healthcare has been and remains complex for many reasons [15–17]. To structure these reasons, barriers hindering the implementation of eHealth have been divided into individual (e.g., motivation or trust), environmental (e.g., financial reimbursement or political barriers), and technical barriers (e.g., security or poor fitting solutions) [18]. Categorizing barriers and the level of impact per stakeholder group (e.g., nurses, doctors or hospital management) helps pinpoint specific problems. Mentioning and measuring the size of a barrier per stakeholder group could further contribute to designing targeted solutions to cope with strained healthcare resources and enhance implementation in orthopedic trauma surgery.

During the COVID-19 pandemic, eHealth was rapidly introduced into daily practice, to provide continuation of healthcare during social distancing measures. Three in ten patients visit the Emergency Department due to a musculoskeletal injury, contributing to the large volume of patients requiring in-hospital acute care [19]. Therefore, a substantial degree of organizational changes (e.g., substitution of care in terms of place, mode and frequency) were required to deal with the disruption of daily activities in orthopedics and traumatology during the COVID-19 pandemic.

One of the eHealth solutions that was introduced to cope with social distancing measures in orthopedic trauma surgery during the COVID-19 pandemic was the Direct Discharge protocol. This protocol includes twelve low-complex musculoskeletal injuries that received routine outpatient follow-up and a cast after their initial Emergency Department visit. With DD, patients receive no routine outpatient follow-up, and a sling or a brace. After extensive information, patients receive the Virtual Fracture Care app (VFC). The VFC app is a self-care application and contains audiovisual support, red flags, and a helpline for questions. The app has been implemented in over 30 hospitals since the start of the COVID-19 pandemic, contrasting with the usual slow implementation in this field. DD has shown to be an effective protocol to significantly reduce secondary healthcare utilization, without compromising patient satisfaction, primary healthcare utilization, and functional outcomes negatively. DD is an example of how eHealth can improve patient outcomes and serve humanity, despite saving resources.

Perhaps that the involuntary exposure to digital alternatives has forced healthcare professionals to overcome ‘individual barriers’ (e.g., motivation or trust) and explore the extent of ‘environmental-’and ‘technical barriers’ in their daily activities and organizations to maintain healthcare accessible during the COVID-19 pandemic.

These forced experiences with multiple types of eHealth (e.g., tele monitoring or Direct Discharge) might have influenced their attitudes toward the usefulness of eHealth in daily practice. In the literature, this potentially permanent paradigm shift has previously been mentioned as ‘the new digital normal’ but the level of penetration of this shift has not been mentioned [20–22]. Even though adoption of eHealth occurred rapidly during the early stages of the COVID-19 pandemic, sustainable use of eHealth is not self-evident and reasons for non-use should be mentioned too. In the current literature no particular studies have focused on individual motivations for non-use of eHealth in orthopedic trauma surgery, but have been performed for major surgery cases [14]. In this study, participants reported that eHealth could diminish the personal interaction with patients, and may not be useful for all patients (and healthcare professionals) due to a lack of digital literacy [23]. Additionally, an important and underreported factor in preventing abandonment of eHealth is the perceived usefulness of innovation among involved healthcare providers (e.g., nurses or doctors) [24]. Assessing the attitudes of healthcare professionals to eHealth is important to increase adoption and prevent abandonment of eHealth. The aim of this study was therefore to survey the use of eHealth in daily practice, perceived opportunities of eHealth in Dutch (orthopedic) trauma surgery, perceived implementation barriers, and desired functions and features amongst healthcare providers working in (orthopedic) trauma surgery.

## Methods

### Study design

A cross-sectional, web-based survey was sent out among the 605 members of the Dutch Society of Trauma Surgery (NVT) and related healthcare professionals on the attitudes toward the usefulness of eHealth in daily practice between November 4, 2021, and March 31, 2022. The survey was introduced at the first physical annual meeting after social distancing after the third COVID-19 pandemic wave in the Netherlands. Healthcare professionals working in orthopedic trauma surgery were eligible for inclusion. Healthcare professionals that did not complete the survey were excluded from final analysis. eHealth was defined as an emerging field in the intersection of medical informatics, public health and business, referring to health services and information delivered or enhanced through the Internet and related technologies. In a broader sense, the term characterizes not only a technical development, but also a state-of-mind, a way of thinking, an attitude, and a commitment for networked, global thinking, to improve health care locally, regionally, and worldwide by using information and communication technology [5]. eHealth is considered a part of digital health, which has been defined as the use of information and communication technologies in medicine and other health professions to manage illnesses and health risks and to promote wellness. This study has been approved by the local scientific and medical ethical committee of the St. Antonius Hospital (P22.010).

### Population and setting

The web-based survey was used to collect a convenience sample of healthcare professionals working in orthopedic surgery and Traumatology in the Netherlands. We distinguished three groups of healthcare professionals physicians (e.g., (orthopedic) surgeons, surgical residents, and emergency medicine doctors), nurses (e.g., operating room nurses or emergency medicine nurses), and other healthcare professionals (e.g., plaster technicians). Plaster technicians handle immobilization materials, and advice patients regarding recovery. Healthcare professionals in orthopedic trauma surgery work in trauma centers (level 1, 2, and 3) which refers to the number of trauma patients per year, injury severity and type of resources available to treat these patients in these centers. Level-1 means tertiary facility care for every aspect of injury. Level-2 means the ability to initiate definitive care for all injured patients. Level-3 refers to the ability to provide prompt assessment, resuscitation, surgery, intensive care and stabilization of injured patients and emergency operations. Healthcare professionals treating patients with traumatic injuries in all hospital settings could fill out the survey (e.g., level-2 trauma center or private clinic).

### Recruitment and consent

The link to a web-based survey was distributed through a QR-code via flyers and presentations at the 2021 Dutch Traumatology Congress, the congress website, and the NVT newsletter after the congress. If participants only partially filled out the survey, a reminder was sent automatically by the RedCap program seven days after the initial start. This was not traceable for the researchers. The survey was closed on March 31, 2022. The disclaimer stated that all participants provided digital informed consent for the anonymous use of their data for open-source publication. For this study, a waiver was obtained by the local scientific and medical ethical committee of the St. Antonius Hospital (P22.010). This study was performed in accordance with the declaration of Helsinki and the required ethical guidelines of BMC Health Services. The demographic data of participants of the congress and the NVT were requested but were not provided due to privacy regulations. The response rate could not be calculated, as solely the number of members of the NVT was shared.

### Survey items

The survey (Appendix A) consisted of five sections with 42 questions, including close-ended questions, multiple-choice questions, 5-point Likert Scales (with 1 meaning absolutely not and 5 meaning absolutely), Visual Analogue Scales (VAS), and free-text questions. The survey included the following sections: (1) demographics: Age, occupation, type of trauma center, sex, smartphone ownership, amount of patients treated with a self-care application instead of follow-up, expected percentage of patients which would prefer digital follow-up over face-to-face follow-up. (2) Current use of eHealth in daily practice: Changed attitude towards usefulness of eHealth after the COVID-19 pandemic, Electronic Patient Record (EPR) use, patient portal use, type of data collection in EPR, usage of telemonitoring, type of applications integrated in EPR. (3) Opportunities and attitudes of eHealth in daily practice: Related to a positive attribution to daily activities, healthcare costs, patient satisfaction, patient information, the burden of care, and on-demand care. (4) Implementation barriers of eHealth: Related to scientific evidence, financial support during implementation, long-term financial support, demand from patients, digital literacy among patients, demand from healthcare professionals, and complex integration of external applications in local EPRs, data protection, law and regulations, and (5) relevance of functions and features of eHealth in Orthopedic- and trauma surgery: Related to automated reminders, timely information describing recovery processes, additional injury-specific information, immobilization-related information, behavioral rules during recovery, telemonitoring, direct communication (e.g., patient to doctor), analgesic-related information, free of surcharge, privacy conditions. At the end of the survey a free-text field was available for additional opportunities, barriers, and functions and features. This survey was based on questions developed by German medical experts in digitization and survey development [25, 26]. These questions were adjusted, translated to Dutch, and checked by a Dutch research center specialized in healthcare innovation research (THINC) [27]. We pretested the survey with five medical professionals to improve clarity. eHealth was defined as any form of digital care that helps deliver and organize health services and information using the internet and related technologies [28]. Patient portals were defined as a mean by which patients can access their health information, including diagnostic test results [29]. Respondents were able to review and change their answers through a ‘back button’. RedCap has an automated function which performs a completeness check and requires confirmation after completion of the survey. The CHERRIES checklist, with accompanied answers has been added in Appendix B.

### Statistical analysis

All data were collected using a web application for building and managing surveys and databases, RedCap [30]. Completed questionnaires were exported, and all statistical analyses were conducted using IBM Statistical Package for the Social Sciences (SPSS), version 27, IBM Corporation [31]. Baseline characteristics were reported descriptively using numbers and proportions for categorical variables, and mean with standard deviation (SD) or median with interquartile range (IQR) as appropriate for numerical variables. For sections opportunities and attitudes of eHealth in daily practice, implementation barriers, and relevance of functions and features, the mean of the 5-point Likert scales was used to rank answers within sections from highest mean to lowest mean. Pearson chi-square tests were used to determine whether there was a statistically significant association between the variables. Statistical significance was considered as an alpha less than 0.05.

## Results

### Demographics

In total, 129 orthopedic surgery and traumatology healthcare professionals started the survey. Eighteen responders did not complete the survey and were excluded from the final analysis. Of the 111 included healthcare professionals, 59/111 (53%) of the responders were male, and the median age was 40 years (IQR 26 to 67). Almost all participants owned smartphones (109/111, 98.2%). The majority of healthcare professionals worked in level-2 trauma centers (88/111, 79%), followed by level-3 trauma centers (12/111, 11%) and level-1 trauma centers (11/111, 10%). Current occupations of participants were (orthopedic) surgeon (37/111, 33%), surgical resident (39/111, 35%), emergency medicine doctor (10/111, 9%), plaster technician (14/111, 13%), others (10/111, 9%), or missing (1/111, 1%) (Table 1). Other occupations were PhD-candidate of a surgical department (*n* = 7), in-hospital project manager (*n* = 2), and Operating Room Nurse (*n* = 1).

**Table 1:** Baseline characteristics of survey participants regarding the usefulness of eHealth in the Netherlands

Variable	Frequency; *n* (%)
*Median age in years (IQR)*	40 (26 to 67)
Below 30 years	27 (24%)
30 to 39 years	40 (36%)
40 to 49 years	22 (20%)
50 to 59 years	15 (13%)
Over 59 years	3 (3%)
Missing	4 (4%)
*Male sex*	59 (53%)
*Smartphone ownership*	109 (98%)
*Current function*	
Surgeon	37 (33%)
Surgical resident	39 (35%)
Emergency medicine doctor	10 (9%)
Plaster technician	14 (13%)
Other	10 (10%)
Missing	1 (1%)
*Working location*	
Academic level-1 Trauma Center	8 (7%)
Level-1 Trauma center	3 (3%)
Level-2 Trauma Center	88 (79%)
Level-3 Trauma Center	12 (11%)

### Current use of eHealth in daily practice

All participants worked with an Electronic Patient Record (EPR) in daily practice (111/111, 100%). The majority of the EPRs had an integrated patient portal (92/111, 83%). Reported types of applications integrated with EPRs were video consultation (11/111, 10%), doctor-to-patient communication (8/111, 7%), and digital questionnaires prior to outpatient follow-up (16/111, 14%), or none (11/111, 10%). Types of data collected through an EPR were pain scores (11/111, 10%), injury symptoms (7/111, 6%), Patient Reported Outcome Measures (PROMs) (26/111, 23%), and Patient Reported Experience Measures (PREMs) (8/111, 7%). Most participants use a computer (41/111, 37%), followed by a smartphone (17/111, 15%), telephone (16/111, 14%), and tablet (11/111, 10%) to communicate digitally with patients.

### Opportunities and attitudes of eHealth in daily practice

**Fig. 1:**
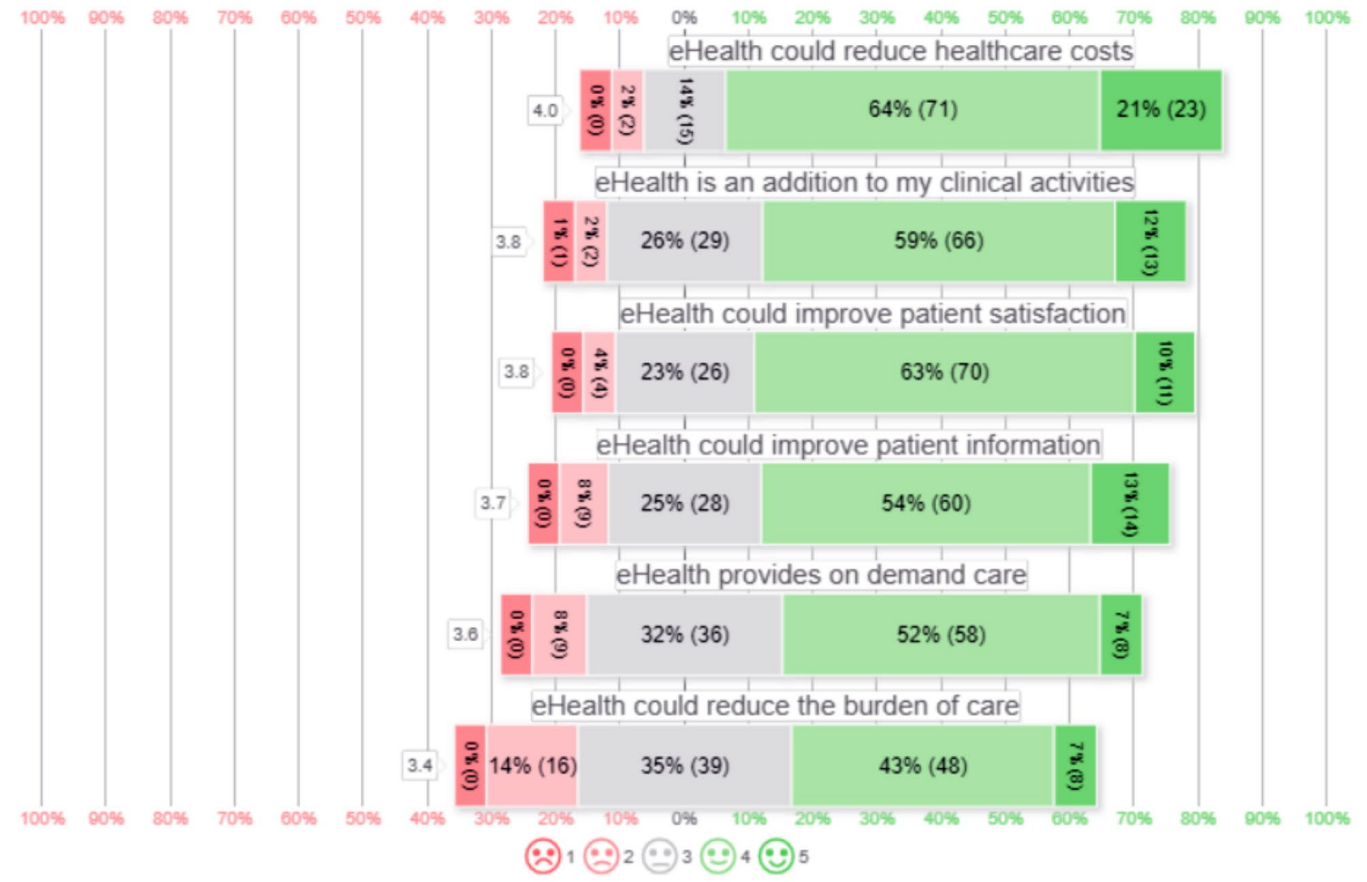
5-point Likert scale distribution of eHealth opportunities in orthopedic surgery and traumatology. □ contains mean of all 5-point Likert scale. 5-point Likert scale 1 meaning fully disagree to 5 meaning fully agree. ( ) contains the absolute number of patients scoring this value

The distribution of eHealth opportunities measured in 5-point Likert scales are presented in Fig. 1.

**Fig. 2:**
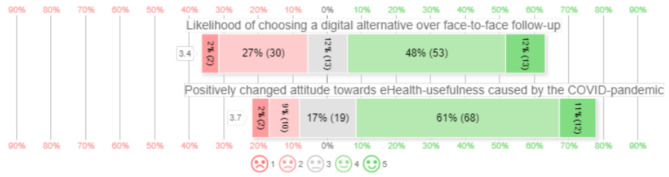
5-point Likert scale describing perceieved usefulness of eHealth in orthopedic surgery and traumatology. □ contains mean of all 5-point Likert scale, distributed in percentage positive and negative responses. 5-point Likert scale 1 meaning fully disagree to 5 meaning fully agree. ( ) contains the absolute number of patients scoring this value

Opportunities for eHealth were scored with positive values in all categories by at least 50% of all answers. The majority of the respondents stated that eHealth could: reduce healthcare costs (94/111, 85%), improve patient satisfaction (81/111, 73%), attributes positively to their clinical activities (79/111, 72%), improve patient information (74/111, 67%), provide 24/7 on-demand care (66/111, 59%), and reduce the burden of care for healthcare professionals (56/111, 50%). The distribution of attitudes towards eHealth measured in 5-point Likert scales are presented in Fig. 2. Most participants reported that the COVID-19 pandemic has influenced their attitude towards the usefulness of eHealth positively 80/111, 72%, 19/111 (17%) were neutral, and 12/111 (11%) reported that it has not influenced their attitude positively. 66/111, (60%) of all participants would choose a digital alternative over face-to-face follow-up if proven a safe technology. Participants expected that 64% of all patients would prefer a digital self-care application over face-to-face follow-up at the outpatient clinic.

### Implementation barriers of eHealth

**Fig. 3:**
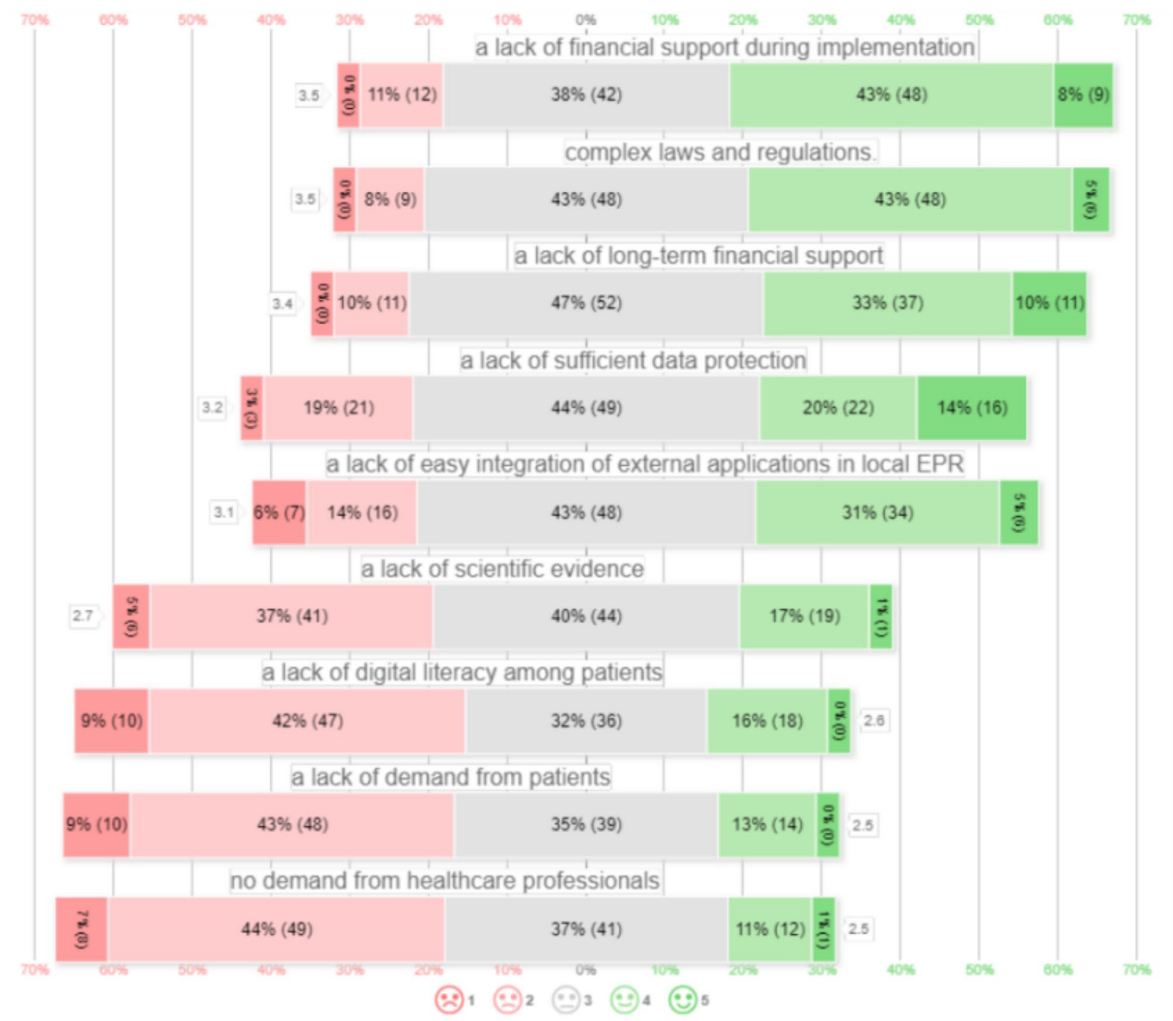
5-point Likert scales of implementation barriers of eHealth in orthopedic surgery and traumatology. □ contains mean of all 5-point Likert scale, with results ordered from most hindering to least and distributed in positive and negative percentage of responses. 5-point Likert scale 1 meaning fully disagree to 5 meaning fully agree. ( ) contains the absolute number of patients scoring this value

The distribution of eHealth opportunities measured in 5-point Likert scales is presented in Fig. 3, arranged from most important barriers to least important barriers. Responders mentioned that in their view eHealth was hindered most by a lack of financial support during implementation (57/111, 51%), followed by complex laws and regulations (54/111, 49%), a lack of long-term financial support (48/111, 43%), complex integration of external applications into local EPR (40/111, 36%), and a lack of sufficient data protection (38/111, 34%). Responders mentioned that in their view eHealth was hindered least by a lack of: demand from healthcare professionals (13/111, 12%), demand from patients (14/111, 13%), digital literacy among patients (18/111, 16%), and scientific evidence (20/111, 18%).

### Relevance of potential functions and features of eHealth in orthopedic surgery and traumatology

**Fig. 4:**
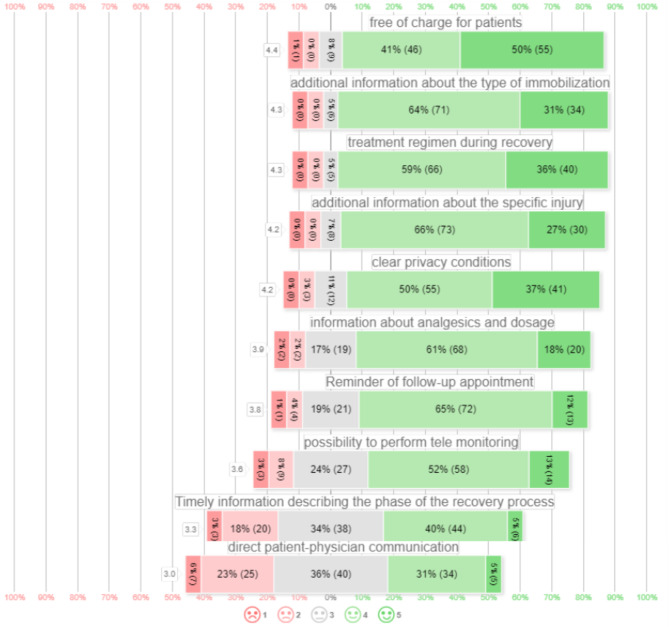
5-point Likert scales of relevance of eHealth in orthopedic surgery and traumatology. □ contains mean of all 5-point Likert scale and scores ordered from most positive to least positive and distributed in positive and negative percentage of responses. 5-point Likert scale 1 meaning fully disagree to 5 meaning fully agree. ( ) contains the absolute number of patients scoring this value

The distribution of potential functions and features measured in 5-point Likert scales are presented in Fig. 4, arranged from most relevant to least relevant functions. Responders stated that most relevant potential functions and features were behavioral rules during recovery (106/111, 95%), followed by additional information about the type of immobilization (105/111, 95%), additional information about the specific injury (103/111,93%), free of charge for patients (101/111, 91%), information about analgesics and dosage (88/111, 79%), clear privacy conditions (96/111, 87%), a reminder of follow-up appointment (85/111, 77%), possibility to perform tele monitoring (72/111, 75%).

Healthcare professionals were less positive regarding the following functions: timely information describing the phase of the recovery process (50/111, 45%), and direct patient-to-doctor communication (39/111, 35%) (Fig. 4). Other functional features mentioned were high usability for all users, such as minorities with different native languages (*n* = 2), nationwide integration with EPR (*n* = 3), the possibility of finalizing the care process (*n* = 2), and red flags during the recovery process (*n* = 1).

## Discussion

Results of this cross-sectional survey show that attitudes of orthopedic surgery or traumatology-related healthcare professionals toward the usefulness of eHealth are positive after the COVID-19 pandemic.

### Comparison to the literature

The results of this study show that all healthcare professionals work with a digital patient portal and EPR. However, the actual use of additional features, such as digital collection of PROMs (23%), remains limited. These results are partly in line with the literature. Multiple British, Iranese, and Brazilian systematic reviews describe an increase in studies regarding eHealth but solely point out the increased number of studies publishing on this topic [32–36]. Despite this increase of publications, epidemiological studies describing eHealth use are lacking. A German survey study that reported ‘actual use’ described similar user rates (79%) of eHealth and smartphone ownership rates (100%) compared to this study but did not report further data of ‘type of use (e.g., PROMs or telemonitoring) [37]. Overall, it can be concluded that there has been an increasing number of studies describing eHealth solutions and reviews describing overall characteristics of these studies but actual use among all potential users per country and specialism are lacking. To improve the knowledge regarding the current condition, nationwide surveys could be performed.

### Attitudes toward eHealth

The changed attitude of healthcare professionals regarding the usefulness of eHealth in daily clinical practice during the COVID-19 pandemic is mostly in line with the literature published after the COVID-19 pandemic. Multiple recent interview and cohort studies performed in Dutch and Flemish healthcare professionals describe that the COVID-19 pandemic has been the turning point for their perceived usefulness of eHealth in clinical practice. Furthermore, these studies describe that eHealth is now an essential complementary addition to healthcare that can decrease the burden of care, increase the accessibility of care, enhance participation, and increase safety for users [38–41]. In these studies, safety of these interventions was mentioned as proper protection of personal data and similar outcomes terms of functional scores or complications. As a comparison to patient attitudes, a recent British scoping review, reported positive patient attitudes regarding eHealth, particularly video consultations. One exception were elderly patients, which preferred face-to-face appointments [42]. When compared to healthcare professional attitudes, data from Uganda, Sweden, and Norway prior to the COVID-19 pandemic, healthcare professionals attitudes varied from skeptical to (very) positive but were reported in small sample sizes, aligning with these findings [43–45].

The high levels of perceived usefulness among both patients and healthcare professional including a positive attitude towards eHealth is interesting, as the actual use remains low and the use of telemonitoring measured in all specialties has slightly decreased over the course of 2022 [46]. This suggests that sustainable adoption of eHealth remains challenging and the return to face-to-face care is imminent. Even though attitudes remain generally positive, eHealth should be a (tested) solution to an existing problem to enhance positive attitudes towards its usefulness after implementation, particularly for those at risk for poor- or non-adoption (e.g., due to low digital literacy). Perhaps the reason for low levels of use have other causes, as the attitudes have been and remain generally positive.

### Opportunities of eHealth in daily practice

Healthcare professionals were positive regarding all the proposed opportunities of eHealth in daily clinical practice. The proposed opportunities were among the top six reported facilitators in the literature up to 2019 [18]. German, Dutch, English and Iranian studies and reviews performed after the era of the COVID-19 pandemic-restriction-measures emphasize on ‘personalized information,’ ‘behavioral rules during recovery,’ ‘the potential to reduce costs, while maintaining high levels of healthcare professionals and patient satisfaction’ as important opportunities [34, 35, 38, 47]. Studies from after the COVID-19 pandemic with a patient perspective report the opportunity of ‘increased self-management’, ‘better healthcare experiences’, ‘better health information’ and ‘more personalized care’ [42, 48, 49]. Even though the results of both healthcare professionals and patient perspective align, patients tend to focus more on their personal relation to care and have less attention for solutions to ‘societal challenges’ such as healthcare costs. In the literature, the types of perceived opportunities are similar compared to this survey, but the level of importance varies. A possible explanation for this variety in importance is that each eHealth intervention is different, designed for a different problem (e.g., shortage of staff, poor accessibility), with different end-users (e.g., patients with low-complex problems versus chronically ill patients), and different settings (e.g., at home, in hospital). It should be mentioned that eHealth can only be implemented in those areas where it solves a problem for particular patients who accept this as an alternative to face-to-face treatment. Major opportunities described in the literature, such as ‘the availability of resources (e.g., financial or staff)’ and ‘early stakeholder involvement’, have been mentioned as antonyms in the barriers sections (e.g., lack of financial resources) [18]. Overall, it can be concluded that the opportunities are perceived similarly among patients and healthcare professionals both from a personal and societal level.

### Implementation barriers of eHealth

Although attitudes have positively changed and potential opportunities are reported widely, barriers to implementing eHealth in daily practice remain. Participants reported that the implementation of eHealth is hindered mainly by a lack of long-term financial support, complex laws and regulation (such as privacy laws), and a lack of short-term financial support. Based on the literature, these barriers can be categorized as ‘environmental’(e.g., lack of financial reimbursement or political barriers) and technical barriers (e.g., security or poor fitting solutions) and have been reported widely in previous studies [18, 38, 47, 50]. ‘Individual’ barriers, such as disbelief or resistance, were not perceived as important in this survey but were an important barrier category in studies published prior to the COVID-19 pandemic [18, 51]. This difference of importance in barrier categories is in line with the literature published after the emergence of the COVID-19 pandemic [38–40]. This difference in perceived barriers is interesting, as personal resistance of Dutch healthcare professionals working in orthopedic- and trauma surgery does not seem to limit the implementation of eHealth anymore. The Direct Discharge protocol is a positive example of an eHealth solution that was implemented during the COVID-19 pandemic in the Dutch orthopedic trauma surgery departments and implemented in over thirty hospitals since [11, 52]. Due to its wide adoption, this example seems to be led by motivation of user and stakeholders based on healthcare professional- and patient experiences [11, 52]. Nevertheless, attitudinal and behavioral changes could also be a result of fear, necessity and anxiety during the COVID-19 pandemic [53]. Therefore, based on our results it unlikely that the accelerated introduction will contribute to the continued use of eHealth innovations as the complexities are persistent and affect new implementation processes. Hospitals and policy makers should gather recommendations made to develop a sustainable strategy for further development of fast and effective implementation of eHealth [54, 55].

### Relevance of potential functions and features of eHealth

In the design of digital tools, the majority of mentioned features were perceived as important. Additional information regarding ‘behavioral rules’, ‘type of immobilization’, ’information regarding specific injuries’, and ‘ no surcharge for patients’ were reported as most important for potential eHealth solutions. The importance of functions and features of eHealth in orthopedic surgery and traumatology is partly in line with the literature. A recent Dutch study underlines the need for reliable information and a prediction of how a patient recovery trajectory is evolving [10]. Dittrich et al. report ‘intuitive usability’ and ‘no advertising’ as the most important features among German (orthopedic) trauma surgeons [56].

Even though the most important features vary per study, these studies underline the importance of excellent usability, and reliable and uniform information regarding immobilization, injury, and recovery in orthopedic surgery and traumatology [10, 56]. In general, there is a paucity of studies focusing on specific demands and needs for eHealth solutions in orthopedic trauma surgery. Potentially studies describing needs or challenges post-injury can be used to enhance acceptance among patients and healthcare professionals, such as qualitative studies during recovery [57].

### Strengths

One of the strengths of this study is the diverse sample of different healthcare professionals included in this study. As reported, (early) stakeholder involvement and engagement is one of the most reported facilitators of implementing eHealth in the literature [18]. This approach underlines that a transformation from face-to-face care physical care to eHealth influences all related stakeholders instead of only patients and doctors. Furthermore, this study describes the use of eHealth tools in daily practice in orthopedic surgery and traumatology departments, which has been underreported in the literature. Another strength is that the study was performed after several COVID-19 pandemic waves, and no new waves occurred during the study period. During the time of inclusion, the COVID-19 pandemic restrictions (e.g., social distancing and lockdowns) were lifted gradually. This allowed healthcare professionals to get used to eHealth solutions and see pros and cons during daily activities less dominated by social distancing.

### Limitations

This study has some major limitations to address. Firstly, the response of 111 participants may not represent all healthcare professionals working in orthopedic surgery and traumatology in the Netherlands, introducing potential selection bias and responder bias. Furthermore, no response rate, page visitor comparison of demographics has been made due to privacy regulations. This may have led to overly positive results. Even though the generalizability of this study is debatable based on this sample, the results are in line with the most recent literature, which includes high-level evidence such as systematic reviews, meta-analyses, and a narrative review. Answers to this observational survey were forced-choice answers but respondents were given the option to add additional free-text comments at the end of the survey. Nevertheless, this design could have overlooked opportunities and barriers which may have been present among healthcare professionals.

### Clinical relevance and generalizability

This study gives insight into the adoption and use of eHealth in Dutch orthopedic surgery and traumatology, which are currently lacking in the literature, particularly in surgical departments. Even though the sample was small and response rates could not be calculated, results were homogenous compared to the literature published after the COVID-19 pandemic, making these results more generalizable. The results of this study are clinically relevant, as they can be used to provide an overview of perceived barriers, opportunities and functions and features. Besides being clinically relevant, these results are also interesting from a political- and organizational perspective, as healthcare professionals are willing to use eHealth but express the need for help with barriers outside their level of influence in daily clinical practice. Unfortunately, due to a low response no sub analysis between stakeholders could be made. In terms of generalizability it is important to mention that most studies published in this field studies setting in middle-to-high or high income countries, making these results only applicable to those countries. It seems considerable that studies in middle to low income countries will lead to different results as a different healthcare setting will lead to different opportunities and barriers. Nevertheless, this should be studies in future studies.

### Future research

Future studies should focus on monitoring eHealth trends and actual use over time to measure the permanent effect of the COVID-19 pandemic on the adoption of eHealth. Identifying risk factors for non-adoption or abandonment and exploring the reasons for the low use of eHealth in daily practice could further optimize eHealth adoption. To overcome the perceived barriers, future studies should also explore perceived barriers on a macro-level (e.g., national governmental level), as these could influence ‘environmental’ or ‘technical’ barriers. This study focused on eHealth attitudes in orthopedic trauma surgery, and did not use a validated standard to measure healthcare acceptance or digital literacy, such as the Technology Acceptance Model (TAM), the Unified Theory of Acceptance and Use of Technology 2 (UTAUT-2), or the eHealth literacy scale ( EHEALS). Using such a uniform tool could help the generalizability and comparability and is adviced to be used in future studies, accompanief with study-specific questions.

## Conclusion

Results of this cross-sectional survey show that attitudes of orthopedic surgery or traumatology-related healthcare professionals toward the usefulness of eHealth are positive after the COVID-19 pandemic.

The majority of healthcare professioansl would use eHealth if proven safe and believes patients would prefer this too. Healthcare professionals report that eHealth could reduce costs, improve satisfaction, improve patient information, and attribute positively to daily clinical activities. Nevertheless, healthcare professionals also report that the implementation of eHealth is hindered mostly by financial, law, and regulatory barriers. Future studies should focus on alleviating persistent barriers so durable implementation of this ‘new digital normal’ becomes feasible.

## Electronic supplementary material

Below is the link to the electronic supplementary material.

Supplementary Material 1

Supplementary Material 2

## Data Availability

The data is available upon request by the authors, and the authors have no competing interests.

## Abbreviations

COVID: 19-Coronavirus Disease 2019
DD: Direct Discharge
NVT: Nederlandse Vereniging voor Traumachirurgie, translated: Dutch Society of Trauma Surgery
VFC: Virtual Fracture Care
QR: Quick-response code
VAS: Visual Analogue Scale
THINC: The healthcare innovation center
SD: Standard deviation
IQR: Interquartile range
SPSS: Statistical package for the Social Sciences
PhD: Doctor of Philosophy
EPR: Electronic Patient Record
PROM: Patient Reported Outcome Measure
PREM: Patient Reported Experience Measure
CEHRES: The Centre for eHealth Research Roadmap
TAM: Technology Acceptance Model
UTAUT: 2-Unified Theory of Acceptance and Use of Technology 2
EHEALS: eHealth literacy scale
