# Supplementary material for: Attitudes toward the adoption of eHealth amongst healthcare professionals in trauma surgery – the new digital normal?
Source: BMC Health Serv Res. 2024 Dec 18;24:1606. doi: 10.1186/s12913-024-11259-7 (PMC11654188; doi:10.1186/s12913-024-11259-7)
Supplement: Supplementary file 1 — Supplementary Material 1 [file 12913_2024_11259_MOESM1_ESM.docx]

**Appendix A.** English translation of the Dutch survey exploring current attitudes of the usefulness of eHealth in daily clinical practice in Orthopedic surgery and traumatology

09-12-2022 10:55 projectredcap.org

***Study ID 53***

*Page 1*

**Barriers and Facilitators in the implementation of eHealth during the COVID-Pandemic in Orthopedics and Traumatology (BAFECT)**

Dear colleague,

The COVID-19 pandemic has forced us to develop alternatives to face-to-face care to continue care during social distancing measures. In multiple types of healthcare this has led to a quick implementation of eHealth. A frequently mentioned argument is that after the COVID-19 pandemic eHealth will remain to play an important role in patient care.

This study aims to gather to which extent digital care fits and is implemented into acute, and outpatient care at the Orthopedic Surgery and Traumatology department. Furthermore the extend of opportunities, and barriers to the implementation of eHealth as reported by Healthcare professionals will be determined.

This questionnaire is part of a feasibility study of the implementation of eHealth and Virtual Fracture Care in the Netherlands. We will use these data for scientific research. By filling out the survey, you consent to the use of an anonymized version of the data for publication. Completing the survey requires approximately five minutes.

Thank you for your participation

Yours sincerely,

Gijs Willinge & Jelle Spierings,

MD, PhD-candidates Virtual Fracture Care

Also on behalf,

Coreteam Virtual Fracture Care

Dr. Ruben van Veen, trauma surgeon OLVG,

Dr. Bas Twigt, trauma surgeon OLVG,

Drs. Marike Kokke, trauma surgeon St. Antonius Hospital &

Dr. Detlef van der Velde, trauma surgeon St. Antonius Hospital

| **Baseline data** | | |
| --- | --- | --- |
| 1. | I filled out this survey on: | (YYYY-MM-DD) |
| 2. | My current occupation is: | - Trauma surgeon - Orthopedic surgeon - Surgeon - Emergency Medicine doctor - Resident ED* - Physician Assistant ED* - Resident orthopedic surgery - Resident surgery - Other |
| 3. | If other, my current occupation is: | (free text) |
| 4. | In what level of trauma center do you work? | - A level-1 trauma center - A level-2 trauma center - A level-3 trauma center |
| 5. | What is your birth year? | (YYYY) |
| 6. | What is your sex? | - Female - Male |

09-12-2022 10:55 projectredcap.org

***Study ID 53***

*Page 3*

**Personal use and attitudes towards eHealth**

The first part of this questionnaire explores the personal use and attitudes towards the usefulness of eHealth in daily practice.

| **Baseline Measurements** | | |
| --- | --- | --- |
| 7. | Do you own a smartphone? | - Yes - No |
| 8. | If it suits the type of treatment, would you treat a patient with a self-care application instead of face-to-face follow-up? | - Never - Sometimes - Neutral - Often - Always |
| 9. | I expect that (*fill out percentage*) of my patients with a traumatic injury would prefer to be treated with a self-care application if it suits the treatment. | 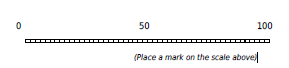 |

09-12-2022 10:55 projectredcap.org

***Study ID 53***

*Page 4*

**Current use of eHealth in daily practice**

The following questions will explore your daily experiences with eHealth in Orthopedic Surgery and Traumatology.

| 10. | To which extend has the COVID-19 pandemic positively changed your attitude towards the usefulness of eHealth. | - Completely not - Not - Neutral - Quite a bit - Completely |
| --- | --- | --- |
| 11. | Does your hospital have an Electronical Patient Record (EPR)? | - Yes - No |
| 12. | Is your EPR supported by a Patient Portal*? | - Yes - No |
| 13. | What type of data is collected through applications in your EPR? | - Pain scores - Registration of symptoms - PROMs^#^ - PREMs^$^ - Other - None |
| 14. | If chosen other, please answer this question:  Our hospital uses remote monitoring systems for: | (*free text field)* |
| 15. | What type of medium do you use for tele monitoring? | - Smartphone - Tablet - Computer - Landline - None |
| 16. | What type of applications have been integrated into your EPR? | - Tele consult (audiovisual consult) - Sending and delivering messages from patient to doctor and vice versa - Digital questionnaires (prior to follow-up) - We do not have additional applications |

- One answer allowed
- Multiple answers allowed

# Patient Reported Outcome Measures

$ Patient Reported Experience Measures

09-12-2022 10:55 projectredcap.org

***study ID 53***

*Page 5*

**Opportunities**

The following statements explore the potential opportunities of eHealth in Orthopedic Surgery and Traumatology. To which extend do you (dis)agree with the following statements? (1 totally disagree to 5 totally agree).

| **Opportunities** | | | | | | |
| --- | --- | --- | --- | --- | --- | --- |
|  |  | *Totally disagree* | *Disagree* | *Neutral* | *Agree* | *Totally agree* |
| 17. | eHealth is a positive attribution to my clinical activities. |  |  |  |  |  |
| 18. | eHealth could reduce healthcare costs |  |  |  |  |  |
| 19. | eHealth could improve patient satisfaction |  |  |  |  |  |
| 20. | eHealth could improve patient information |  |  |  |  |  |
| 21. | eHealth could decrease the burden of care for Healthcare professionals |  |  |  |  |  |
| 22. | eHealth provides 24/7 on-demand care |  |  |  |  |  |

09-12-2022 10:55 projectredcap.org

***Study ID 53***

*Page 6*

**Implementation**

The following questions explore factors that potentially hinder the implementation of eHealth in daily practice.

To which extend do you (dis)agree with the following statements? (1 totally disagree to 5 totally agree).

| **Implementation**  The implementation of eHealth is hindered, due to … | | | | | | |
| --- | --- | --- | --- | --- | --- | --- |
|  |  | *Totally disagree* | *Disagree* | *Neutral* | *Agree* | *Totally agree* |
| 23. | A lack of scientific evidence |  |  |  |  |  |
| 24. | A lack of financial support during implementation |  |  |  |  |  |
| 25. | A lack of long-term financial support |  |  |  |  |  |
| 26. | A lack of demand from patients |  |  |  |  |  |
| 27. | A lack of digital literacy among patients |  |  |  |  |  |
| 28. | A lack of demand from healthcare professionals |  |  |  |  |  |
| 29. | Complex integration of external applications in local Electronical Patient Records |  |  |  |  |  |
| 30. | A lack of sufficient data protection |  |  |  |  |  |
| 31. | Complex laws and regulations |  |  |  |  |  |

***Study ID 53***

*Page 7*

**Functions and Features**

To which extend do you believe the following functions and features are relevant for eHealth in Orthopedic surgery and Traumatology.

| **Functions and Features** | | | | | | | |
| --- | --- | --- | --- | --- | --- | --- | --- |
|  |  | *Totally disagree* | | *Disagree* | *Neutral* | *Agree* | *Totally agree* |
| 32. | Automated reminders of follow-up appointments, |  | |  |  |  |  |
| 33. | Timely information describing the phase of the recovery process |  | |  |  |  |  |
| 34. | Additional information about the specific injury, |  | |  |  |  |  |
| 35. | Additional information about the type of immobilization |  | |  |  |  |  |
| 36. | Behavioral rules during recovery (permissive weight-bearing, driving, swimming etc.) |  | |  |  |  |  |
| 37. | Possibility to perform tele monitoring (e.g., collection of pain scores, or PROMs^#^) |  | |  |  |  |  |
| 38. | Direct patient-physician communication |  | |  |  |  |  |
| 39. | Information and dosage of analgesics |  | |  |  |  |  |
| 40. | Free of charge for patients |  | |  |  |  |  |
| 41. | Clear privacy conditions for patient |  | |  |  |  |  |
| 42. | Additional useful functions or features | | *(free text field)* | | | | |

# Patient Reported Outcome Measures

***Study ID 53***

*Page 8*

**End of Survey**

| **Thank you for your participation!** |
| --- |
